# First detection and molecular characterization of Jingmen tick virus with a high occurrence in Rhipicephalus (Boophilus) microplus collected from livestock in Cameroon (2024)

**DOI:** 10.1186/s13071-025-06670-w

**Published:** 2025-02-05

**Authors:** Paloma Kiwan, Eva Lopez, Morena Gasparine, Geraldine Piorkowski, Agathe Colmant, Achille Paguem, Stephanie Mvodo, Laurence Thirion, Xavier de Lamballerie, Remi Charrel, Alessandra Falchi

**Affiliations:** 1 Unite Des Virus Emergents (UVE: Aix-Marseille Univ, Universita Di Corsica, IRD 190, Inserm 1207, IRBA), Marseille, France; 2 Faculté d’Agriculture et de Médecine Vétérinaire, Université de Buea, Buea, Cameroon; 3 Centre National de Référence des Arbovirus, Marseille, France

**Keywords:** Jingmen tick virus, Ticks, Cattle, Sheep, Cameroon

## Abstract

**Background:**

Jingmen tick virus (JMTV) is a novel tick-borne virus detected for the first time in *Rhipicephalus (Boophilus) microplus* in China. To date, there is no information regarding the circulation of JMTV in ticks collected from livestock in Cameroon. As part of the surveillance for arboviral circulation, this study aimed to assess the presence of JMTV in ticks collected from livestock (cattle and sheep) in an area of the Akonolinga health district, Center Region, Cameroon.

**Methods:**

A cross sectional study was carried out during the dry season between 5 and 14 March 2024. Ticks were collected from cattle and sheep in six sampling sites in an area approximately 30 km long and 18 km wide along the Nyong River, in central Cameroon. Ticks were identified morphologically and molecularly. Total RNA/DNA was extracted from tick pools and screened for JMTV RNA using a segment 2 RT-qPCR system. Positive JMTV pools were sequenced for partial JMTV-Segment 1 and full genome analyses.

**Results:**

A total of 622 ticks, organized into 251 pools were collected from 155 cattle and nine sheep. They consisted of five species covering three genera: *R. (B.) microplus* (472; 75.9%), *Amblyomma variegatum* (118; 19.0%), *Hyalomma truncatum* (13; 2.1%), *Hyalomma rufipes* (2; 0.3%), and other *Rhipicephalus* spp. (17; 2.7%). The quantitative reverse transcription polymerase chain reaction (qRT-PCR) screening of 251 tick pools yielded 61 JMTV-positive pools, of which 58 corresponded to *R. (B.) microplus*. Multiple sequence analysis revealed that JMTV from the Akonolinga area shared > 95% identity with strains from Guinea, and that these strains clustered phylogenetically together.

**Conclusions:**

We provide molecular evidence of the presence of JMTV in *R. (B.) microplus* and *A. variegatum* collected from cattle and sheep from an area not yet recognized as endemic for this virus, confirming its wide geographical distribution.

**Graphical Abstract:**

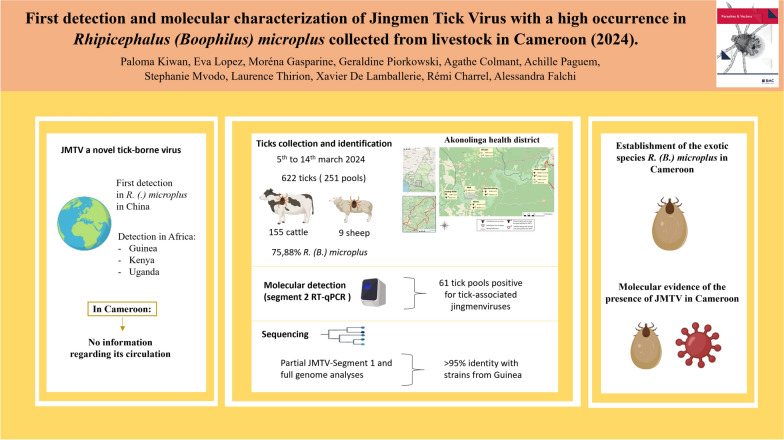

**Supplementary Information:**

The online version contains supplementary material available at 10.1186/s13071-025-06670-w.

## Background

Jingmen tick virus (JMTV) is a novel tick-borne virus characterized by a segmented RNA genome [1]. Jingmen tick virus was first described in *Rhipicephalus (Boophilus) microplus* ticks in the Jingmen region of the Hubei province (China) in 2010. Following this discovery, JMTV has been detected in multiple arthropod and vertebrate species, alongside other viruses genetically related to segmented flavivirus, classified as jingmenviruses, such as Alongshan virus [2]. The partial genetic relatedness concerns non-structural proteins from segments 1 and 3 similar to flaviviruses NS5, NS2B, and NS3. Segments 2 and 4 have a different evolutionary origin and are, to date, specific to jingmenviruses [3, 4].

Jingmenviruses group phylogenetically into two main clades: one clade comprises tick- and vertebrate-associated jingmenviruses, while the other includes insect- and other host-associated jingmenviruses [5].

Although the pathogenicity of JMTV remains poorly understood, the virus has been detected in multiple common species of ticks and in patients presenting with mild to severe febrile disease [6]. Jingmen tick virus was shown to be present and replicate in human skin tissue by in situ hybridization [6]. These results indicate that a potential pathogenicity for humans merits further investigation.

The primary mode of transmission of JMTV is believed to occur through tick bites during bloodsucking. Despite regional variations in the species of ticks reported to harbor JMTV (i.e., *Haemaphysalis longicornis*, *R. sanguineus*, *Ixodes sinensis*, and *Amblyomma javanense*), the most common species in which JMTV RNA has been detected is* R. *(*B.*) microplus [5]. Jingmen tick virus RNA has also been identified in other arthropods, including mosquitoes [5]. Jingmen tick virus RNA and specific antibodies have been detected in mammals such as cattle, bats, goats, and various rodent species [4], as well as in samples derived from primates and reptiles, specifically tortoises [7, 8].

Jingmen tick virus has a wide geographic distribution in ticks: the Americas (Brazil, Colombia, and Trinidad and Tobago), Asia (China, Japan, Laos, and Türkiye), Europe (France, Romania, Finland, and Serbia), and Africa (Kenya and Guinea) [5, 9]. Focusing on Africa, JMTV was initially detected in *R. geigyi* and subsequently in other *Rhipicephalus* spp. collected from domestic cattle in Guinea [10, 11], as well as in *Amblyomma*, *Rhipicephalus*, and *Hyalomma* species collected from ruminants and tortoises in Kenya [8]. In addition to ticks, JMTV has also been detected in non-human primates in Uganda [7]. However, the prevalence and distribution of JMTV in other African countries is still poorly characterized. Until this study, there was no information regarding the circulation of JMTV in ticks collected from livestock in Cameroon. As part of the surveillance of arboviral circulation, this study aimed to assess the presence of JMTV in ticks collected from livestock (cattle and sheep) in an area of the Akonolinga health district, Center Region, Cameroon.

## Methods

### Description of the study sites

**Fig. 1:**
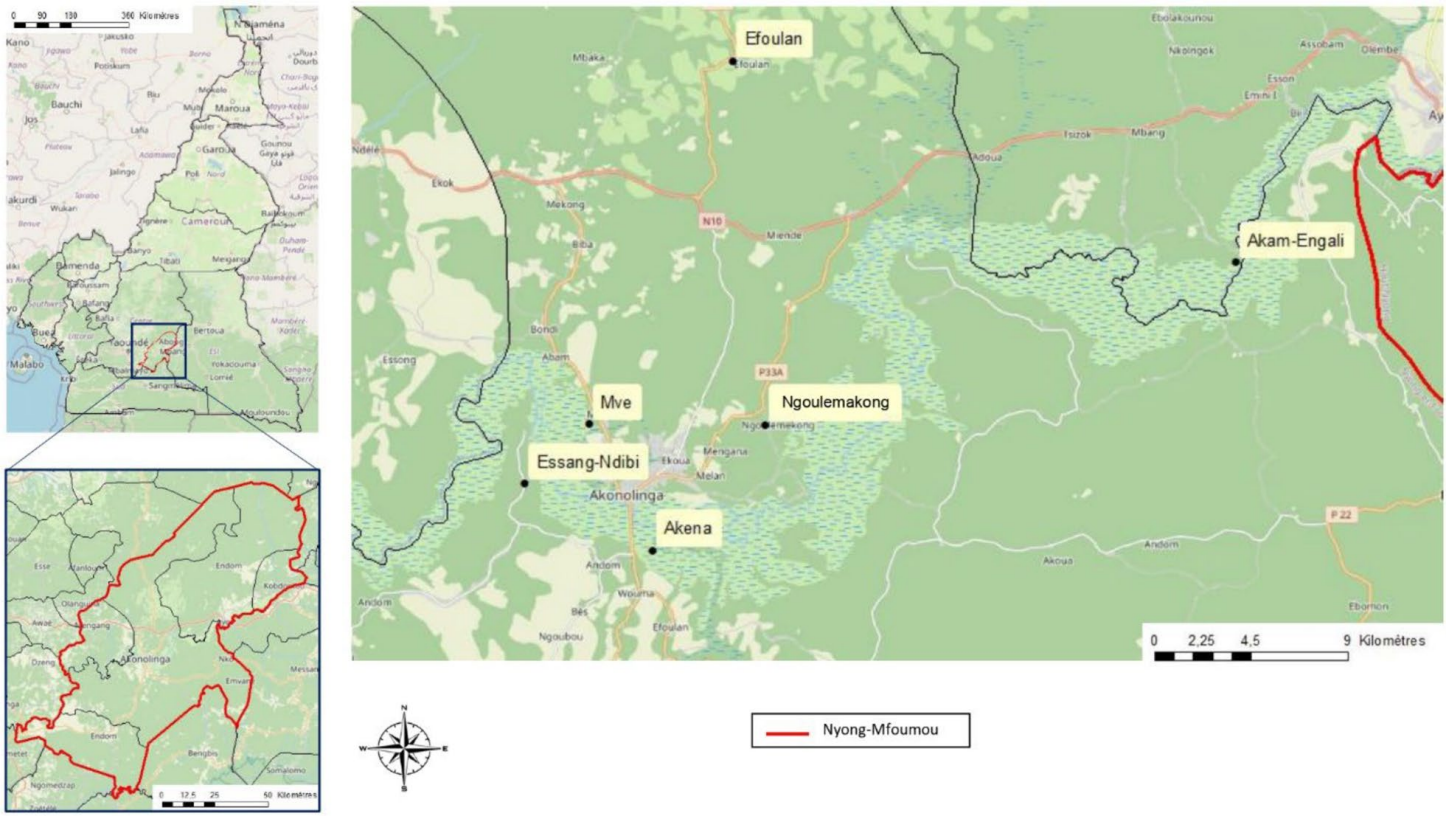
Map of Nyong-Mfoumou department showing the geographical location of the six sampling sites studied in the Akonolinga area

The Akonolinga health district is located in the department of Nyong-Mfoumou, 100 km east of Yaoundé in the Center Region of Cameroon, with a population of 105,789 inhabitants in 2015 (Fig. 1). The Akonolinga area lies within the forest–savannah transitional ecological zone (humid forest–savannah mosaic), thus exposing human and animal populations to both ecosystems. The environmental conditions are influenced by seasonality, as this zone has a subtropical climate with two rainy seasons per year (August–November; April–May) and two dry seasons (December–March and July–August) [12]. The Nyong River Basin in this area, which has several tributaries throughout the villages, is characteristically known for its swampy banks, which is the contact point between human population and domestic and wild animals also providing suitable habitats for many arthropod vectors. The populations are typically rural, and their main activities are farming, fishing, and subsistence livestock rearing (mainly poultry, goats, sheep, and pigs) within close range of human living quarters. In this area, cattle and sheep are raised by the Fulani ethnic group, a pastoral community spanning Central and West Africa [13]. The Fulani graze mainly Zebu White Fulani cattle on extensive communal pastures. Many herders still practice transhumance (seasonal migration) in the dry season along river valleys in search of pasture. The cattle population in the central region of Cameroon is estimated at 276,855 head. In the Akonolinga area included in this study, the cattle population is estimated at 6675 and the sheep population at 1250, which are reared under an open grazing system mainly along the Nyong River (unpublished data communicated by Ministry of Livestock, Fisheries and Animal Industries [MINEPIA]).

### Sampling sites and data collection

Between 5 and 14 March 2024 (dry season), ticks were collected from cattle and sheep in six sampling sites (Efoulan, Akam-Engali, Mvé, Akena, Essang-Ndibi, and Ngoulemakong) in an area approximately of 30 km long and 18 km wide along the Nyong River, nearby the town of Akonolinga, as illustrated in Fig. 1. During the dry season, livestock graze in the riverbed, and during the rainy season, they move to other, less accessible grazing areas.

### Tick collection and morphological and molecular identification

At each site, all cattle and sheep available at the time of the inspection were examined for the presence of ticks (adults and immature stages). Cattle and sheep were restrained and kept standing, and all the body parts of the cattle were examined. Ticks were collected from cattle and sheep using blunt steel forceps and placed inside a collection tube containing 70% ethanol. The samples were sent after obtaining import authorization (number 13205107 from the French Ministry of Agriculture, Food and Forestry) to the virology laboratory of the Unité des Virus Emergents of the University of Corsica. The specimens were identified taxonomically [14], with the help of a veterinary entomologist of the University of Buea (Cameroon), and grouped by genus, sex, and stage, with a maximum of nine individuals by pool and stored at −80 °C for further analysis. To complement morphological identification of ticks, molecular analysis of the partial sequence of the mitochondrial 12S ribosomal RNA (rRNA) gene sequences was performed as previously described [15]. The polymerase chain reaction (PCR) products were examined on an ethidium-bromide-stained 1.5% agarose gel and amplicons of the correct size purified for Sanger sequencing on ABI 3730xl analyzer. All tick pools detected positive for JMTV RNA were selected for 12S rRNA gene sequencing to confirm morphological tick identifications.

### RNA/DNA extraction

Pools of ticks were mechanically homogenized in 1 ml minimum essential medium using the TissueLyser II (QIAGEN) at 5500 rpm for 3 min. Each pool was spiked with a predetermined quantity of MS2 bacteriophage prior to extraction to monitor nucleic acid extraction, reverse transcription, and PCR amplification, as well as to detect any inhibitors and enzymatic reactions [16]. According to the manufacturer’s instructions, total RNA/DNA was extracted from 200 µL homogenates using KingFisher and the MagMAX™ Viral/Pathogen Ultra Nucleic Acid Isolation Kit, eluted in 100 μL of buffer and stored at −80 °C.

### Real-time qPCR of tick pools and JMTV sequencing

All ticks collected were screened for jingmenvirus RNA using a RT-qPCR systems (gTJ-seg2) targeting segment 2 [9], designed to detect multiple jingmenvirus sequences, including JMTV, Yanggou tick virus (YGTV), Alongshan virus (ALSV), and *Pteropus lylei* jingmenvirus (PLJV). These viruses all belong to the tick-associated jingmenvirus group. Each amplification reaction was prepared in a 25 µL final volume per tube and comprised 3 µL nucleic acids, 1.25 µL sense and antisense primers, 12.5 µL 2 × buffer, 0.5 µL reverse transcriptase/Taq polymerase, and 6.5µL RNAse free water. The thermal cycle used was as follows: (i) 55 °C −10 min; (ii) 98 °C −2 min; and (iii) 40 cycles consisting of 98 °C −10 s, 55 °C −10 s, 68 °C −1 min 15 s and a final step of 68 °C −5 min. All tick pools that tested positive in the initial screening described above were subsequently tested using three primer pairs targeting JMTV segment 1 (nt 627–2545; Supplementary Material 1) [3]. The amplification reactions were carried out on a conventional PCR system (2720 thermal cycler) using the SuperScript™ IV One-Step RT-PCR System kit (ThermoFisher ref: 12594100). The amplified products were purified (NEBLabs ref: T3010) and pooled in equimolar proportion. The pooled fragments were then sequenced using the MinIon platform following the manufacturer’s instructions (Oxford Nanopore ref: SQK-LSK 109 and NBD 96). A random two-step RT-PCR was performed on the RNA extract (Merck ref: WTA2) from one JMTV-positive tick pool with a low cycle thresholds (Ct), following the manufacturer’s instructions [9]. Subsequent sequencing of the amplified RNA was carried out using the Ion Torrent S5 platform (Thermo Fisher Scientific), according to the manufacturer’s instructions.

### Sequence and phylogenetic analyses

#### 12S rRNA gene sequences and phylogenetic analyses

To confirm the identity of each 12S rRNA gene sequences (tick species), the sequences were compared with those available in the GenBank database using the BLASTn program [17]. Sequences obtained were aligned with published sequences. Trees were constructed using the maximum likelihood with the Tamura–Nei model (best DNA/protein model [ML]) in MEGA11 software, with 500 bootstraps [18]. The tick pools selected to identify 12S rRNA gene sequences were those showing JMTV-positive results.

#### JMTV gene sequences and phylogenetic analyses

Reads were trimmed and assemblies were performed using Geneious Prime 2024.0.7 using the closest reference sequence to our data, determined using NCBI BLASTn [17]. For JMTV, a full genome sequence from Guinea was used as reference (Genbank accession numbers MK673133–MK673136). The most closely related sequences identified with BLAST, as well as reference JMTV sequences, were used in a multiple nucleotide sequence alignment performed with multiple sequence comparison by log-expectation (MUSCLE) on Geneious Prime 2024.0.7. The alignments obtained were used to infer maximum likelihood phylogenies in MEGA11, with the Tamura–Nei model for the 12S tick sequences and the general time reversible (GTR) substitution model for JMTV sequences, with equilibrium frequencies ML optimized and a fixed (0.0) proportion of invariable sites as determined by Smart Model Selection in PhyML [19], and with 500 bootstraps.

### Statistical analysis

A 95% confidence interval (CI) was employed to estimate the individual-level prevalence of pathogens, on the basis of the assumption that each PCR‐positive pool contained at least one positive tick. The estimation was conducted using the pooled prevalence model available on the EpiTools epidemiological calculator platform [20]. Categorical variables were described using numbers and percentages, with comparison using chi-squared test or Fisher’s exact test when appropriate. Missing values were excluded from analysis. If the *P*-value was ≤ 0.05, it was considered statistically significant. All the analyses were computed with R software, version 4.0.3 (4.0.3, R Core Team, 2021, R Foundation for Statistical Computing, Vienna, Austria; https://www.r-project.org/**).** For the spatial analysis, the exact site of each tick found attached to cattle and sheep was recorded. Each location was transferred into ArcMap v.10.8.2 software (ArcGis Desktop) and plotted on maps.

## Results

### Tick infestation rates

**Fig. 2:**
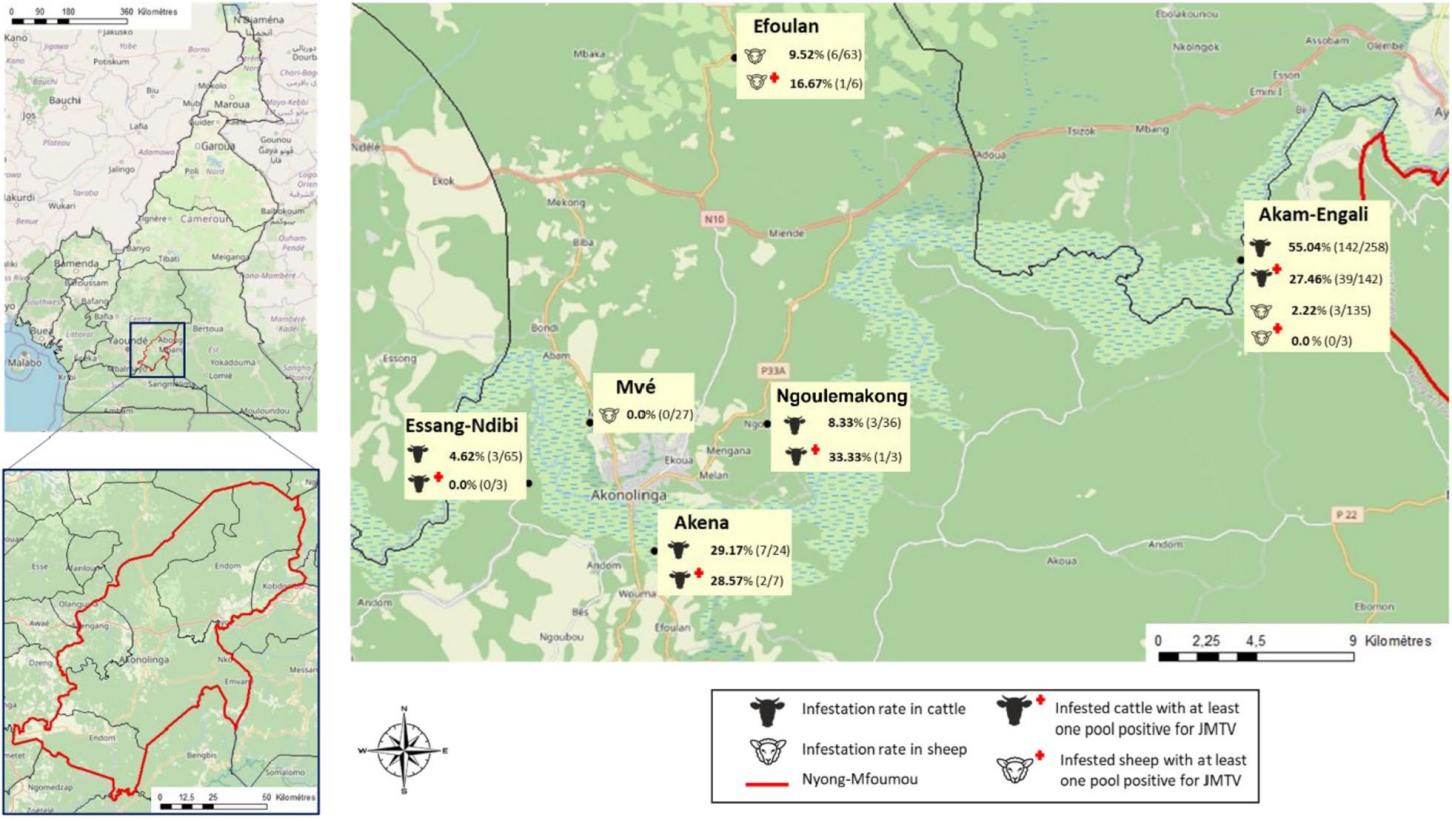
Map of Nyong-Mfoumou department showing the geographical location of the six sampling sites studied in the Akonolinga area and the percentage (%) of infested animals, both cattle and goats, and the percentage of animals with at least one JMTV-positive tick pool at each location

A total of 383 cattle and 225 sheep were examined for ticks (Table 1; Fig. 2). Among the 383 cattle, 67.4% (*n* = 258) were sampled in Akam-Engali, 9.4% (*n* = 36) in Ngoulemekong, 6.3% (*n* = 24) in Akena, and 17.0% (*n* = 65) in Essang-Ndibi. Among the 225 sheep, 60.0% (*n* = 135) were sampled in Akam-Engali, 28.0% (*n* = 63) in Efoulan, and 12.0% (*n* = 27) in Mvé (Fig. 2). The infestation rate was higher in cattle (40.5%; 155 out of 383) than in sheep (4.0%; 9 out of 225; *P* < 0.001). The overall infestation rate in cattle and sheep was 27.0% (164 out of 608), with the most infested cattle in Akam-Engali (55.0%; 142 out of 258) and the most infested sheep in Efoulan (9.5%; 6 out of 63; Fig. 2).

**Table 1:** Rates of tick infestation by animal species and sampling sites

Sites	Status	Cattle	Sheep	Total
Akam-Engali	Examined	258	135	393
	Infested	142 (55.0%)	3 (2.2%)	145 (36.9%)
Ngoulemakong	Examined	36	0	36
	Infested	3 (8.3%)	0	3 (8.3%)
Akena	Examined	24	0	24
	Infested	7 (29.2%)	0	7 (29.2%)
Essang-Ndibi	Examined	65	0	65
	Infested	3 (4.6%)	0	3 (4.6%)
Efoulan	Examined	0	63	63
	Infested	0	6 (9.5%)	6 (9.5%)
Mve	Examined	0	27	27
	Infested	0	0 (0.0%)	0 (0.0%)
Total	Examined	383	225	608
	Infested	155(40.5%)	9 (4.0%)	164 (27.0%)

### Morphological and molecular identification of ticks

A total of 622 ticks, organized into 251 pools containing an average of 4 ticks (range 1–9 ticks), were collected from 155 cattle (589 ticks organized into 237 pools) and from 9 sheep (33 ticks organized into 14 pools). The overall adult male-to-female ratio in the collected ticks was 0.47:1 (193 males, 411 females), with a ratio of 0.46:1 (179 males, 392 females) in cattle and of 0.74:1 (14 males, 19 females) in sheep. Because of engorged status and/or absence of some morphological criteria, some *Rhipicephalus* spp. were not identified to the species level. The 622 ticks collected comprised four main species in three genera: *R. (B.) microplus* (472; 75.9%), *Amblyomma variegatum* (118; 19.0%), *Hyalomma truncatum* (13; 2.1%), *H. rufipes* (2; 0.3%), and other *Rhipicephalus* spp. (17; 2.8%; Table 2). In cattle, the predominant tick species was *R. (B.) microplus* (453 out of 589; 76.9%), followed by *A. variegatum* (105 out of 589; 17.8%). Similarly, in sheep, *R. (B.) microplus* was the most common tick species (19 out of 33; 57.6%) followed by *A. variegatum* (13 out of 33; 39.4%; Table 2).

**Table 2:** Percentage of distribution of tick species among cattle and sheep

Tick species (*N* = 622)	*n*	%
*Rhipicephalus (B.) microplus*	472	75.9
*Cattle*	453	76.9
*Sheep*	19	57.6
*Amblyomma variegatum*	118	19.0
*Cattle*	105	17.8
*Sheep*	13	39.4
*Hyalomma truncatum*	13	2.1
*Cattle*	13	2.2
*Sheep*	0	0.0
*Hyalomma rufipes*	2	0.3
*Cattle*	2	0.3
*Sheep*	0	0.0
*Rhipicephalus* spp.	17	2.7
*Cattle*	16	2.7
*Sheep*	1	3.0
Overall cattle	589	94.7
Overall sheep	33	5.3
*Total*	622	

### Phylogenetic analysis of *R. (B.) microplus*

**Fig. 3:**
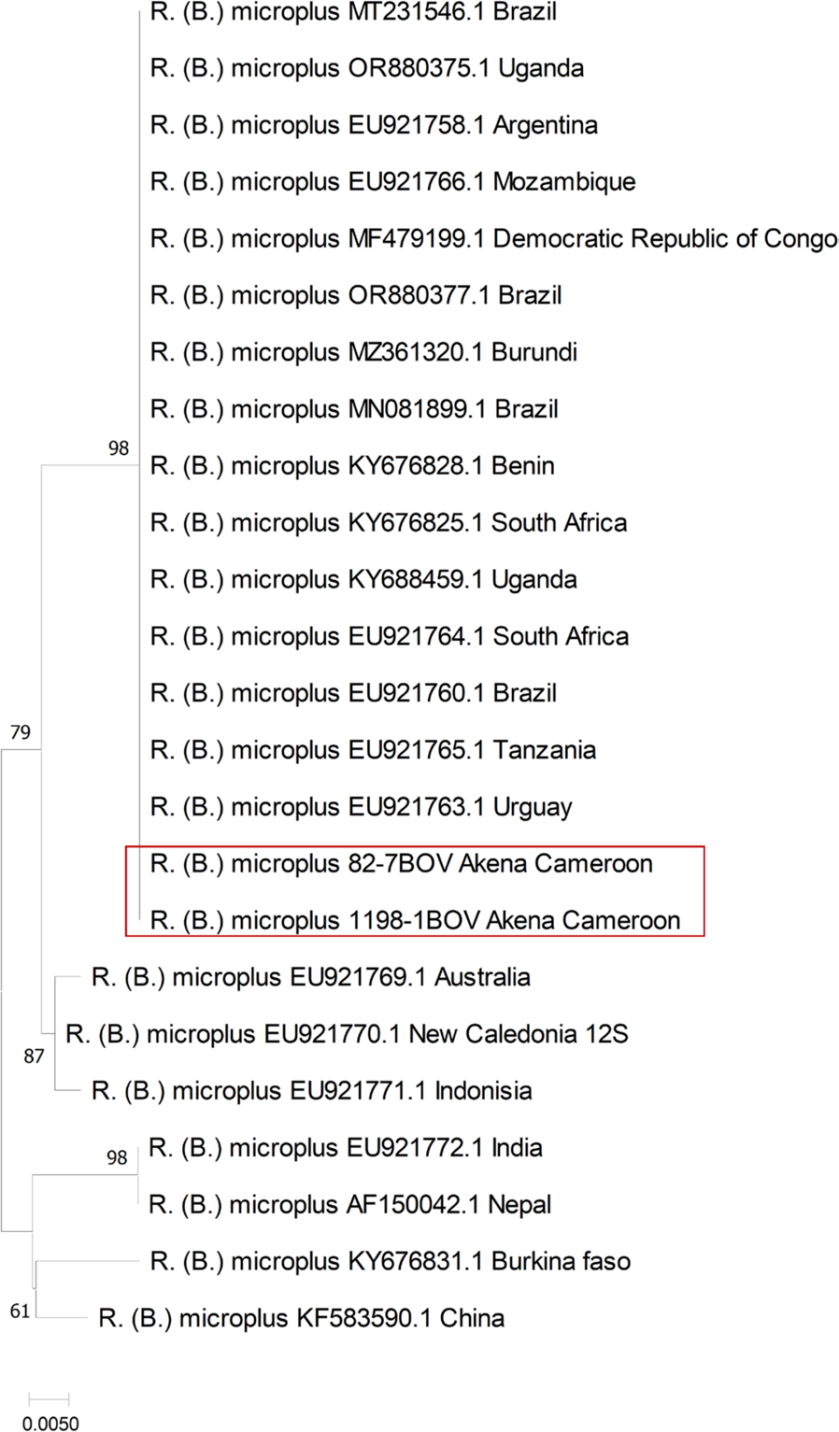
The phylogenetic tree was built on the basi of the 310-bp fragment of the 12S rRNA gene of *R. (B.) microplus* using the maximum composite likelihood method. This analysis involved 24 nucleotide sequences. The percentage of replicate trees in which the associated taxa clustered together in the bootstrap test (500 replicates) is shown next to the branches. Evolutionary analyses were conducted in MEGA11 [18]. The sequences were analyzed using the Tamura–Nei (TN) substitution model. The scale bar indicates the number of substitutions per site

Among the 163 tick pools classified as *R. (B.) microplus* on the basis of morphological criteria, 38 were further validated through amplification and sequencing of a partial fragment of their 12S rRNA gene (Fig. 3). All the 38 12S rRNA segment sequences detected in *R. (B.) microplus* were 100% identical; thus, two sequences were used for the construction of the tree. These sequences displayed nucleotides (nt) identities ranging from 96% to 100% with *R. (B.) microplus* detected in other regions worldwide. These sequences were from a monophyletic clade with sequences reported in Uruguay (EU921763.1), Tanzania (EU921765.1), Brazil (EU921760.1; MT231546.1; OR880377; MN081899.1), South Africa (EU921764.1; KY676828.1), Argentina (EU921758.1), Uganda (KY688459.1; OR880375.1), Mozambique (EU921766.1), Democratic Republic of Congo (MF479199.1), Burundi (MZ361320.1), and Benin (KY676828.1), with 100% nt identity.

### Detection of RNA JMTV in ticks collected from animals

The qRT-PCR screening of 251 tick pools yielded 61 tick-associated jingmenvirus–positive pools (Table 3). One pool (ID 642-1) consisting of a single individual male of *R. (B.) microplus* tick, collected from one sheep (16.6%; 1 out of 6 sheep) in the Efoulan region, tested positive for tick-associated jingmenvirus RNA. The pooled prevalence in sheep was 3.0% (95% CI 0.2–12.7%). The other 60 positive tick pools belonged to *R. (B.) microplus* at 95.0% (*n* = 57), to *A. variegatum* at 33.3% (*n* = 2), and to *Rhipicephalus* spp. at 1.6% (*n* = 1) and were collected from cattle. Specifically, 47 pools were collected from 39 cattle from Akam-Engali, 12 pools from 2 cattle from Akena, and 1 pool from 1 cow or bull from Ngoulemakong, with respective prevalence of cattle with positive ticks of 27.5% (39 out of 142), 28.6% (2 out of 7), and 33.3% (1 out of 3; Table 3; Fig. 2). The pooled prevalence of tick-associated jingmenvirus RNA in cattle was 12.1% (95% CI 9.4–15.2%), with a value of 16.3% (95% CI 12.7–20.6%) in *R. (B.) microplus* tick pools and 1.9% (95% CI 0.3–5.8%) in *A. variegatum* tick pools. One pool consisted of nymphs (*Rhipicephalus* spp). The pooled prevalence was 12.9% [95% CI 9.6–16.2%] in male tick pools (*n* = 15) and 9.2% [95% CI 5.5–14.1%] in female tick pools (*n* = 44).

**Table 3:** Characteristics of JMTV-positive tick pools in cattle and sheep

Animal	Sample site	Animal ID	Tick pool ID	Pool size (number of ticks/pool)	Tick species	Sex	Ct
Cattle	Akam-Engali	1113	1113_2^*^	1	*R. (B.) microplus*	Female	27
		1100	1100–1^*^	9	*Rhipicephalus* spp.	Nymphe	19
		1091	1091–1	1	*R. (B.) microplus*	Female	28
		1117	1117–2^*^	1	*R. (B.) microplus*	Female	19
		1053	1053–1	6	*R. (B.) microplus*	Male	24
			1053–3	1	*Amblyomma variegatum*	Male	22
			1053–4	1	*R. (B.) microplus*	Female	22
		1097	1097–1	1	*R. (B.) microplus*	Male	22
		1161	1161–1	6	*R. (B.) microplus*	Female	35
		1096	1096–1^*^	4	*R. (B.) microplus*	Male	24
		1189	1189–1	2	*R. (B.) microplus*	Female	31
		422	422–1	4	*R. (B.) microplus*	Female	31
		8	8_2	5	*R. (B.) microplus*	Female	21
		426	426–1^*^	6	*R. (B.) microplus*	Female	26
			426–2^*^	3	*R. (B.) microplus*	Male	21
		44	44–1	4	*R. (B.) microplus*	Female	30
		1169	1169–1	3	*R. (B.) microplus*	Female	20
		45	45–1	4	*R. (B.) microplus*	Female	27
		1150	1150–2^*^	1	*R. (B.) microplus*	Female	23
		1023	1023–1^*^	4	*R. (B.) microplus*	Female	28
		354	354–2	3	*R. (B.) microplus*	Female	34
		1168	1168–1^*^	5	*R. (B.) microplus*	Female	28
		1025	1025–1^*^	5	*R. (B.) microplus*	Female	21
			1025–2	4	*R. (B.) microplus*	Female	38
		1015	1015–1^*^	5	*R. (B.) microplus*	Female	23
		424	424–1^*^	6	*R. (B.) microplus*	Female	25
		1195	1195–1^*^	4	*R. (B.) microplus*	Female	24
		141	141–1^*^	2	*R. (B.) microplus*	Female	20
		1109	1109–1^*^	6	*R. (B.) microplus*	Female	24
		181	181–2	4	*R. (B.) microplus*	Male	23
		172	172–1^*^	5	*R. (B.) microplus*	Male	20
		1137	1137–1	6	*R. (B.) microplus*	Female	27
			1137–2	2	*R. (B.) microplus*	Male	32
			1137–3^*^	7	*R. (B.) microplus*	Female	17
		428	428–1	3	*R. (B.) microplus*	Female	40
		176	176–1^*^	1	*R. (B.) microplus*	Female	19
		1198	1198–1^*^	5	*R. (B.) microplus*	Female	22
		161	161–1	2	*R. (B.) microplus*	Male	37
			161–2^*^	1	*R. (B.) microplus*	Female	20
		178	178–2^*^	1	*R. (B.) microplus*	Male	23
		17	17_1^*^	2	*R. (B.) microplus*	Male	17
		171	171–1^*^	7	*R. (B.) microplus*	Male	24
			171–2	2	*Amblyomma variegatum*	Male	33
		1054	1054–1^*^	3	*R. (B.) microplus*	Male	22
		167	167–2^*^	3	*R. (B.) microplus*	Female	20
		357	357–1	6	*R. (B.) microplus*	Female	31
		1042	1042–2^*^	1	*R. (B.) microplus*	Female	23
	Akena	92	92–1	1	*R. (B.) microplus*	Female	38
			92–3	1	*R. (B.) microplus*	Female	34
			92–6^*^	1	*R. (B.) microplus*	Female	20
			92–7	2	*R. (B.) microplus*	Female	23
			92–9^*^	4	*R. (B.) microplus*	Female	22
		82	82–1	6	*R. (B.) microplus*	Female	32
			82–2	6	*R. (B.) microplus*	Female	30
			82–3	6	*R. (B.) microplus*	Female	28
			82–4	6	*R. (B.) microplus*	Female	19
			82–5	6	*R. (B.) microplus*	Female	22
			82–6^*^	6	*R. (B.) microplus*	Female	19
			82–7^*^	6	*R. (B.) microplus*	Female	17
	Ngoulemakons	580	580–1	6	*R. (B.) microplus*	Male	27
Sheep	Efoulan	642	642–1	1	*R. (B.) microplus*	Male	33

^*^Confirmed to be JMTV by sequencing

Out of the 42 cattle with at least one JMTV-positive pool, 40.5% (*n* = 17) had all their ticks pooled in one sample, while 59.3% (*n* = 25) had ticks divided into multiple pools, ranging from 2 to 10 tick pools. Of the cattle with multiple tick pools, 20.0% (5 out of 25) had all pools positive to JMTV, while 80.0% (20 out of 25) had a mix of positive and negative pools. The percentage of JMTV-positive pools per animal are shown in Supplementary Table 2.

### Phylogenetic analyses of JMTV based on partial S1 sequences

**Fig. 4:**
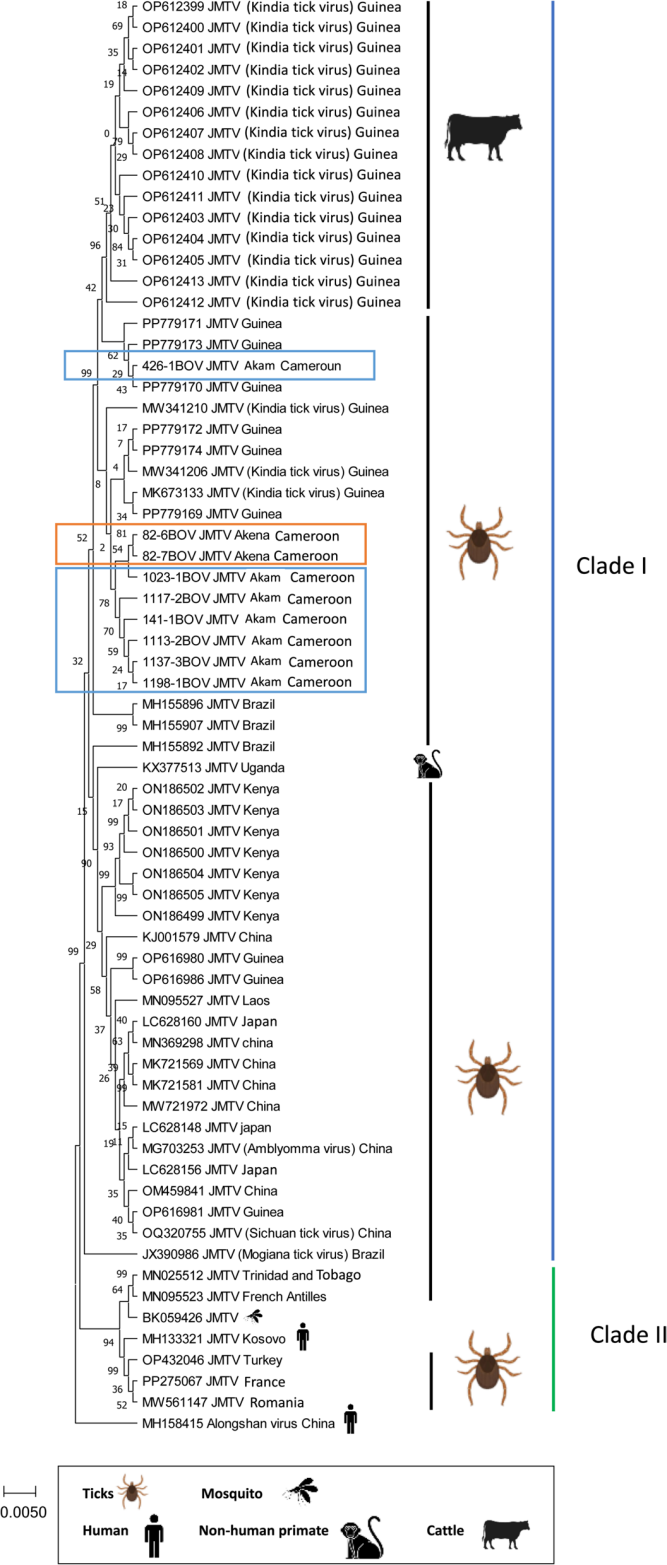
The phylogenetic tree was built on the basis of the 1256-bp fragment of the NSP1 gene using the maximum composite likelihood method. This analysis involved 68 nucleotide sequences. The percentage of replicate trees in which the associated taxa clustered together in the bootstrap test (500 replicates) is shown next to the branches. Evolutionary analyses were conducted in MEGA11 [18]. Sequences were analyzed using the GTR model method. The sequences generated in the present study are highlighted in blue and orange. Alongshan virus sequence (strain H3 MH158415) was used as the outgroup. The scale bar indicates the number of substitutions per site

We obtained usable partial sequences (1256 nucleotides) of segment 1 for 30 of the 61 JMTV-positive tick pools collected from 29 cattle (26 tick pools from 25 cattle from Akam-Engali and 2 tick pools from 2 cattle from Akena). We identified and removed identical sequences, selecting nine representative sequences to include in the phylogenetic analysis. Partial sequences obtained in this study showed an average nucleotide identity of 99.21%. These sequences were most closely related to and clustered phylogenetically with JMTV strains from Guinea, within clade I, which includes sequences from Africa, Asia, and South America (Fig. 4). Our sequences from Cameroon showed (i) 86.7–98.8% nt identity (average 97.6%) to JMTV detected ticks from Guinea; (ii) 89.7–90.8% (average 90.2%) nt identity to JMTV from strains identified in Kenya; and (iii) 89.0–89.6% (average 89.3%) nt identity to JMTV strain from Uganda.

### Full genome sequences

**Fig. 5:**
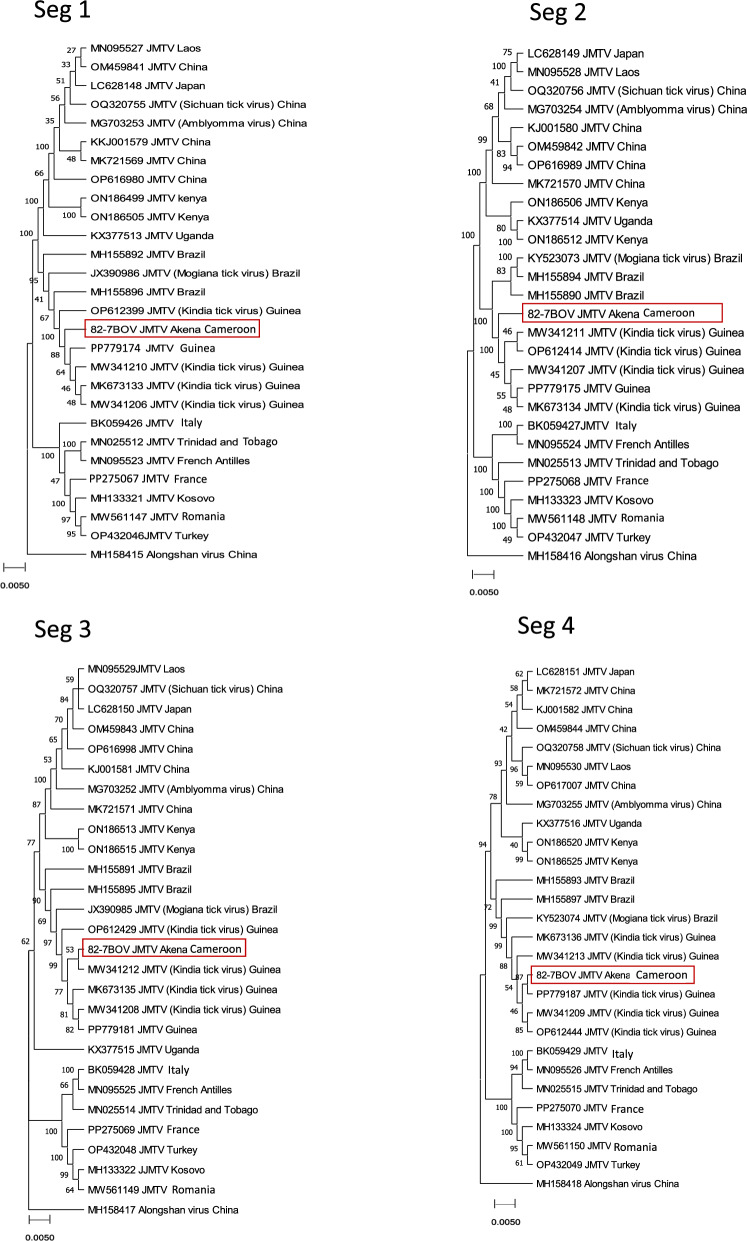
Phylogenetic trees were built for each segment using the maximum composite likelihood method. The analyses were based on a total of 28 sequences for each segment. Phylogenetic trees were inferred from MUSCLE alignments using MEGA11 software [18]. All segments were analyzed using the GTR model. The analyses included 500 bootstrap replicates to assess tree robustness, with values expressed as percentage branch labels. Alongshan virus sequences (strain H3 MH158415–MH158418) were used as the outgroup. The scale bar represents the number of substitutions per site

The JMTV-positive tick sample (82-7BOV) from the Akena site was selected among the positive JMTV positive samples due to its low Ct and good sequence quality. Direct next generation sequencing (NGS) provided partial nucleotide sequences of the four segments as: S1 2961 bp, S2 2812 bp, S3 2670 bp and S4 2725 bp. All four segments of JMTV were closely related to JMTV sequences detected in ticks from Guinea with nt identity ranging from 97% to 99% (Fig. 5). The sequences of the four segments of JMTV from the present study also showed close nt identity with the strains of JMTV detected in Brazil with nt identity ranging from 89% to 98%. Compared with other JMTV genomes reported in Africa, the JMTV sequences obtained in this study share nt identity of 85–90% with JMTV strain RC27 detected in plasma of primates in Uganda and 86–89% with JMTV strains detected in ticks collected in Kenya (Fig. 5).

## Discussion

Here, we report for the first time the detection of JMTV mainly in *R. (B.) microplus* collected during the dry season from cattle and sheep in a forest–savannah transitional ecological zone of Akonolinga, in central Cameroon.

*R. (B.) microplus*, primarily parasitizing cattle, is widespread in tropical and subtropical regions, including India, Malaysia, China, Central and South America, and Australia, where it has been present for decades. It subsequently spread to Southern, East, and West Africa and was first reported in Cameroon in 2019 [21, 22]. This species was likely introduced through animals purchased from areas where this tick is already present, and while its spread was historically slow, this process appears to have accelerated in recent years [23]. Here, *R. (B.) microplus* was the predominant tick species found on cattle and sheep. This is congruent with data reported in the Menoua and Noun division of the West Region of Cameroon [23]*.* However, *R. (B.) decoloratus* was described as the most prevalent species in studies conducted in Cameroon, with a relative prevalence of 62.2% reported in a study from March 2012 to February 2013 [24], and 30.25% in a study conducted in 2019–2020 [25]. Nevertheless, our data suggested the replacement of the native species *R. (B.) decoloratus* with the exotic species *R. (B.) microplus*. A similar observation was reported in the Soutpansberg region, Limpopo province in South Africa [23]. The replacement of *R. (B.) decoloratus* by *R. (B.) microplus* may be due to *R. (B.) microplus* having a shorter biological cycle and higher egg production capacities and its larvae surviving on vegetation during winter, giving it a reproductive advantage over *R. (B.) decoloratus* [26]. In addition to these biological factors, environmental factors such as insecticide use may also play a role. *R. (B.) microplus* may have been selected for resistance to acaricides, while *R. (B.) decoloratus* populations could have been reduced by acaricide treatments, allowing resistant *R. (B.) microplus* to persist and spread [26]. The high abundance of this tick in our study confirms the ongoing circulation of *R. (B.) microplus* in central Cameroon and indicates the progressive establishment of this species in the region.

*R. (B.) microplus* is widely recognized as the most significant ectoparasite and vector of livestock diseases [25]. Additionally, its resistance to safe and affordable pyrethroid insecticides represent a serious problem for endemic countries, as it complicates efforts to control tick populations and the spread of diseases affecting animals [27]. It is known to spread various animal pathogens, including *Babesia bigemina*, *B. bovis*, and *Anaplasma marginale* [22], and has been associated with the transmission of JMTV, first identified in this tick species in China in 2010 [1]. Since then, JMTV has been detected in *R. (B.) microplus* across several countries, such as French Antilles, Brazil, Colombia, and Trinidad and Tobago [4]. In this study, JMTV was primarily detected in *R. (B.) microplus* ticks. This species, originally native to South and Southeast Asia, is thought to have spread to Madagascar and East Africa in the nineteenth century through cattle transport, before later reaching South America [28]. Phylogenetic analysis of these ticks showed that the 12S rRNA gene is highly conserved, with tick sequences from this study identical to one another and to those previously reported from Brazil, Uruguay, Tanzania, South Africa, Argentina, Uganda, Mozambique, Democratic Republic of Congo, Burundi, and Benin. The similarity of our sequences with those from Brazil could be explained by the movement of *R. (B.) microplus* from Brazil to West Africa through the cattle trade, and subsequently from West Africa to Central Africa, particularly to Cameroon, which is located along a major transboundary cattle trade route between these two regions. The phylogenetic results also suggest that the tick might have been introduced to Cameroon from Southern and eastern and western Africa, where the invasion of this species has been extensively documented [22]. However, the exact timing and manner of its introduction to central Cameroun remain unclear.

In comparison to previous similar studies investigating JMTV in ticks collected from ruminants, the JMTV pooled prevalence observed in the present study, estimated as individual-level prevalence of JMTV, was in agreement with prevalence values reported in ticks ranging from 53% to 63% in China, 25% to 67% in Brazil, 6% to 46% in Trinidad and Tobago, and 24% to 77% in the French Antilles [5]. A recent study conducted in Corsica (France) reported a minimum infection rate (MIR) of 0.54% in ticks (mainly *R. bursa*) collected from cattle [9]. In Kenya, detection rates of 0.0%, 0.3%, and 1.8% were observed in cattle, goats, and sheep, respectively [8]. Additionally, a 2021 study in Guinea identified JMTV with a prevalence of 2.8% in ticks collected from livestock [11]. The positive samples for which no sequences were obtained cannot be definitively classified as JMTV, as the detection method used also amplifies ALSV, PLJV, and YGTV. Although the coexistence of different jingmenviruses in the same ecosystem is theoretically possible, there is no evidence of this to date, making it more likely that these sequences correspond to JMTV. [5].

JMTV has been detected in various tick species, primarily within the *Rhipicephalus* spp. and including other genera such as *Amblyomma*, *Dermacentor*, *Haemaphysalis*, *Hyalomma*, and *Ixodes* [5]. In our study, we found that, for most animals with positive tick pools, not all tick pools from the same animal tested positive for JMTV. While we cannot draw definitive conclusions about the roles of the ticks and the animals in the transmission of JMTV, these observations suggest that the ticks themselves may carry the virus independently of the animal’s viremia, raising the possibility that positive ticks could act as vectors for JMTV transmission. However, the exact role of these tick species in JMTV transmission remains unclear and requires further investigation.

The genomic sequences of a new jingmenvirus species were identified in a human sample from Cameroon in a study conducted between 2015 and 2019. Phylogenetic analysis revealed that this virus clustered with insect-associated jingmenviruses and is phylogenetically distinct from JMTV, which belongs to the tick-associated jingmenviruses [29]. In our study, the JMTV sequences belong to the African–Asian–South American clade I that is distinct from the Caribbean–European clade II. This finding is congruent with the geographic allocation reported so far with JMTV. JMTV Cameroon sequences are most closely related to those from Guinea [10]; interestingly, Guinea and Cameroon are 2500 km apart. An important feature of JMTV epidemiology is the apparent existence of long-distance dispersals. As it is unlikely for ticks themselves to move over long distances, the most plausible route is human-mediated transportation of infected cattle and sheep [1]. The JMTV sequences identified in Kenya and Uganda, although still classified within the same clade, demonstrate a higher degree of genetic variation when compared with those from Guinea and Cameroon. Before drawing definitive conclusions, we need to consider the absence of epidemiological studies on JMTV and jingmenviruses in general, in particular in Africa. A better idea of the dispersion dynamics of JMTV could be obtained by performing similar studies in countries located between Guinea and Cameroon, for instance. In Cameroon, where cattle farming is a vital resource for about 30% of the rural population, the well-structured livestock trade network facilitates cross-border animal movement, increasing the likelihood of JMTV dispersals, especially to neighboring regions such as Gabon, Congo, and Equatorial Guinea [30].

Three points were highlighted in this study: first,* R. *(*B.*) microplus was the main tick collected from cattle and sheep, suggesting the replacement of the native species *R. (B) decoloratus* with the exotic species *R. (B.) microplus*; second, JMTV RNA was detected in almost 30% of *R. (B.) microplus* tick pools collected from cattle, suggesting that this species plays a major role in the transmission of JMTV; third, JMTV strains from Cameroon clustered within a clade closely related to JMTV detected from ticks collected from cattle in Guinea (West Africa).

## Conclusions

To the best of our knowledge, we provide here the first work in Cameroon and Central Africa with detection of JMTV in ticks collected from cattle and sheep. JMTV is an emerging virus that is globally endemic, with the potential to cause epidemiological outbreaks. Additional epidemiological studies, including antibody testing in livestock and humans, are required to evaluate the possible health risks associated with JMTV in the study region. The establishment of the exotic species *R. (B.) microplus* in Cameroon raises concerns about potential outbreaks of various animal pathogens transmitted by this tick. Increased vigilance and enhanced surveillance are crucial to prevent and manage potential animal disease outbreaks associated with this tick in the region.

## Supplementary Information

Additional file 1. Conventional PCR primers for amplifying the S1 segment sequences of JMTV.

Additional file 2. Percentage of JMTV pool positivity per cow/bull.

## Data Availability

Data will be available on request by email to the corresponding author. All the nucleotide sequences were deposited in GenBank under accession numbers PQ478049–PQ478060 for JMTV genome sequences and PQ480081–PQ480082 for *R. (B.) microplus* sequences (12 S rRNA).

## Abbreviations

JMTV: Jingmen tick virus
NGS: Next-generation sequencing
qRT-PCR: Quantitative reverse transcription polymerase chain reaction
